# Case report: Rapid clinical improvement in acute exacerbation of MuSK-MG with efgartigimod

**DOI:** 10.3389/fimmu.2024.1401972

**Published:** 2024-06-07

**Authors:** Geke Zhu, Yongbo Ma, Han Zhou, Xiangtao Nie, Wenjing Qi, Lei Hao, Xiuming Guo

**Affiliations:** Department of Neurology, The First Affiliated Hospital of Chongqing Medical University, Chongqing, China

**Keywords:** myasthenia gravis, MuSK, exacerbation, lymphoplasmapheresis, efgartigimod

## Abstract

Myasthenia gravis with positive MuSK antibody often involves the bulbar muscles and is usually refractory to acetylcholinesterase inhibitors. For MuSK-MG patients who experience acute exacerbations and do not respond to conventional treatments, there is an urgent need to find more suitable treatment options. With the advent of biologic agents, efgartigimod has shown promising results in the treatment of MG. We report a 65-year-old MuSK-MG patient who presented with impaired eye movements initially, and the symptoms rapidly worsened within a week, affecting the limbs and neck muscles, and had difficulties in chewing and swallowing. Lymphoplasmapheresis did not achieve satisfactory results, but after a cycle of efgartigimod treatment, the patient’s symptoms gradually improved and remained in a good clinical state for several months.

## 1 Introduction

Myasthenia gravis (MG) is a chronic autoimmune disease characterized by the production of autoantibodies, which attack the neuromuscular junction on the postsynaptic membrane (1). The most commonly targeted autoantibodies are against the acetylcholine receptor (AChR). In addition, antibodies against muscle-specific tyrosine kinase (MuSK), low-density lipoprotein receptor-related protein 4 (LRP4), and ryanodine receptor (RyR) have also been found to be involved in the pathogenesis of MG (2). Immunological activation caused by complement and antibody deposition leads to postsynaptic damage, resulting in partial or generalized skeletal muscle weakness and extreme fatigue. The symptoms often exacerbate after physical activity, severely impairing the patient’s quality of life. Current treatment strategies for MG are typically based on immunosuppression or immunomodulation (3, 4). However, finding a targeted, well-tolerated, and long-term beneficial treatment option has been a challenge for neurologists, especially for MG patients who are unresponsive to conventional therapies, even including intravenous immunoglobulin (IVIG) or plasma exchange (PE).

With the advent of targeted biologic agents, MG treatment has entered a new era (5). Efgartigimod, the first approved and marketed neonatal Fc receptor (FcRn) antagonist in the world, has shown promising results. In a multicenter, double-blind, randomized, placebo-controlled phase III clinical trial involving 167 patients with generalized MG (6), the Efgartigimod group showed a notable reduction in IgG, AChR antibodies, and Myasthenia Gravis Activities of Daily Living (MG-ADL) scores as early as the first week. The MG-ADL responser (who had at least a 2-point improvement in MG-ADL score) rate reached 68% (44/65), significantly higher than the 30% (19/64) in the control group. Nowadays, Efgartigimod has been approved and marketed in several countries (7), and in most of these countries, its indication specifically stating its use for generalized MG in patients who test positive for AChR (8).

However, currently there is still a lack of clinical experience in the treatment of MuSK-MG, particularly in the management of acute exacerbations. Efgartigimod may potentially serve as a viable treatment option in such cases. Here we report a case of a 65-year-old male patient with MuSK-MG. He initially presented with ocular muscle involvement manifested as impaired eye movements. The symptoms rapidly progressed within a week, and he did not respond well to symptomatic treatment or lymphoplasmapheresis (LPE). Eventually, after a cycle of Efgartigimod treatment, the symptoms were effectively controlled.

## 2 Case presentation

Our patient gradually developed oculomotor disturbances without any obvious triggers one month before admission. These disturbances eventually progressed to complete immobility of the eyes, accompanied by dizziness. He also had a history of hypertension and diabetes.

Neurological examination revealed complete restriction in bilateral ocular upward and downward gaze, lateral gaze, and medial gaze. It is worth noting that there was no ptosis, but the fatigue test of the levator palpebrae superioris muscle was positive. All other physical examinations were normal. Extensive laboratory investigations were performed (complete blood count, urine analysis, liver and renal function, electrolytes, coagulation profile, homocysteine levels, lipid profile, blood glucose levels, thyroid function tests, antinuclear antibody spectrum, antineutrophil cytoplasmic antibody spectrum, cerebrospinal fluid routine and biochemistry) with normal results. Nerve conduction studies showed no significant abnormalities in the motor and sensory conduction velocities of the limbs. However, low frequency repetitive nerve stimulation test of bilateral facial nerve and accessory nerve produced positive results. Subsequently, the neostigmine test was performed and yielded a positive result (with significant improvement in eye movements after neostigmine injection). Chest CT scan showed no thymoma. Myasthenia gravis was diagnosed (Myasthenia Gravis Foundation of America I, MGFA I), with a Quantitative Myasthenia Gravis (QMG) score of 4 (although the patient had no diplopia subjectively, considering the inability to move the eyes, we assigned the most severe score of 3 for this item; ptosis: score 1). Therefore, symptomatic treatment was initiated with pyridostigmine bromide (180mg/day). However, in the following days, the patient rapidly developed limb weakness, neck muscle weakness, and difficulties with swallowing and chewing. The QMG score increased to 20, and the MG-ADL score was 9 (MGFA IIIa). Serological immune testing indicated a positive result for MuSK antibodies (>12.00 U/mL, tested via enzyme linked immunosorbent assay, reference value <0.40 U/mL). In response to the acute worsening of the patient’s condition, we immediately started LPE (once every 3 days, a total of three times). After LPE, the patient’s swallowing improved, but limb weakness worsened. QMG score: 21, MG-ADL score: 9 (MGFA IIIa).

**Figure 1:**
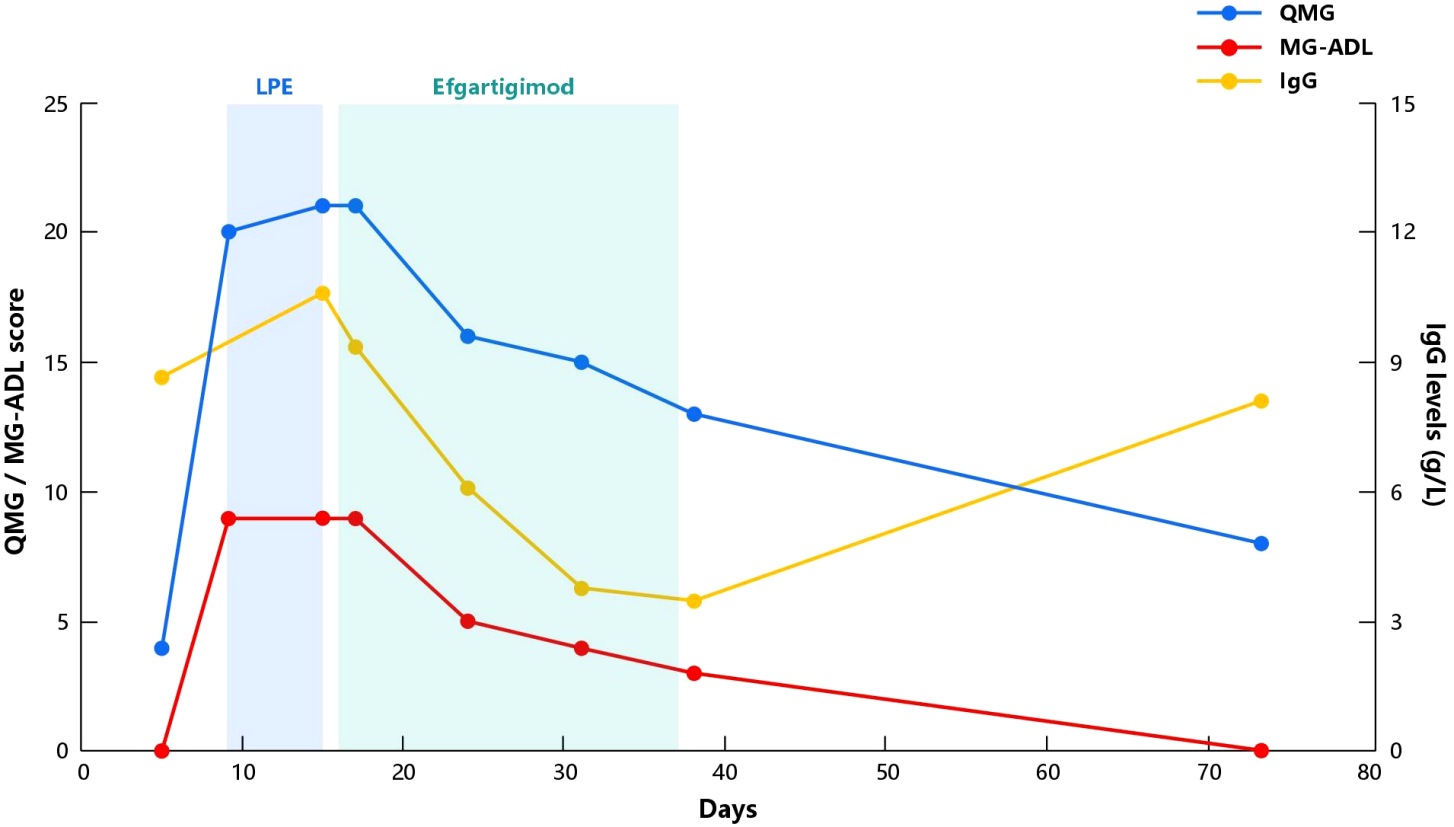
Evolution of QMG score, MG-ADL score and IgG levels in our patient. Day 0 represents the day of hospital admission. LPE, Lymphoplasmapheresis; QMG, Quantitative Myasthenia Gravis; MG-ADL, Myasthenia Gravis Activities of Daily Living; IgG, Immunoglobulin G.

The rapid deterioration of the disease, limited effectiveness of treatment, and the discomfort caused by the invasive procedure of LPE had left the patient feeling extremely disheartened. Corticosteroids are the standard treatment for MuSK-MG, but the patient and his families were wary of its potential side effects. Furthermore, there is a lack of evidence and effectiveness of non-steroidal immunosuppressants for MuSK-MG. Both conventional treatment options mentioned above are unlikely to provide rapid relief. So, after comprehensive consideration, we initiated efgartigimod treatment the day after the third LPE and completed one treatment cycle (10mg/kg, administered once weekly for a total of four weeks). During the four infusion periods, the patient’s eye movements gradually improved, chewing function gradually returned to normal, and overall muscle endurance remarkably improved. IgG levels, QMG and MG-ADL scores showed a clear downward trend (**Figure 1**). Meanwhile, we also monitored his complete blood count, albumin, lipid profile, as well as liver and renal function. All the results were stable and maintained in the normal range, and the patient did not experience any acute infection symptoms such as fever or cough throughout the entire treatment period. One month after the final infusion, the QMG score had reduced to 8, and the MG-ADL score was 0 (MGFA IIa). However, IgG levels started to rise again (**Figure 1**). Currently, the patient has stopped taking pyridostigmine bromide due to gastrointestinal side effects and is not taking any other medication but is temporarily maintaining a good clinical condition.

## 3 Discussion

Approximately 5% of patients with myasthenia gravis can test positive for MuSK antibodies (9), which have a different pathogenesis compared to the most common AChR antibodies. MuSK-MG is usually associated with more severe clinical symptoms, primarily affecting bulbar muscles, leading to difficulties in swallowing, chewing, and speaking, and can progress rapidly within a short period of time (10, 11). Our patient initially presented with ocular muscle involvement, but not the typical ptosis and diplopia in myasthenia gravis. Instead, there were disturbance in his eye movement. Therefore, originally, we considered Miller-Fisher syndrome and cranial neuropathy caused by diabetes within our differential diagnosis. Nonetheless, the positive finding in fatigue test of the levator palpebrae superioris muscle during the physical examination led us to lean towards the diagnosis of myasthenia gravis. Subsequently, low frequency repetitive nerve stimulation test and neostigmine experiment further supported our suspicion. After considering the diagnosis of ocular myasthenia gravis, we initially attempted to control the symptoms with pyridostigmine bromide. Nevertheless, one week after admission, the patient’s symptoms acutely worsened, and serological testing eventually confirmed the patient’s diagnosis as MuSK-MG. It is well-known that MuSK-MG often shows poor response to acetylcholinesterase inhibitors, and conventional doses of pyridostigmine bromide often lead to side effects (12), as was the case with our patient. Additionally, the rapid involvement of the limbs and bulbar muscles in our patient aligns well with the characteristic of rapid progression in MuSK-MG. Fortunately, our patient’s respiratory muscles were not affected.

Corticosteroids are the standard treatment for MuSK-MG, but higher doses are typically required to elicit a response (13). Due to the concerns of our patient and his families about the side effects, we were unsure if the patient would respond to corticosteroids. PE and IVIG are classic non-specific immunotherapies used in cases of acute exacerbation or severe conditions such as myasthenic crisis. PE is typically more effective for MuSK-MG (11–13). LPE is a new therapy that combines lymphocyte exchange with traditional PE. Compared to PE, it not only removes pathological immune factors such as autoantibodies and cytokines from the plasma but also eliminates immune response cells. In recent years, studies have confirmed that LPE exhibits clinical efficacy for MG that is comparable to or even superior to traditional PE, with fewer treatment sessions (14). As previously described, the LPE regimen was one treatment every three days for a total of 1–3 sessions (14–16). Therefore, three sessions of LPE should be sufficient for our patient. Unexpectedly, our patient did not show significant improvement with LPE during the acute exacerbation phase. Coupled with his severe anxiety and depression, there was an urgent need for newer and more effective treatment methods for our patient. Coincidentally, efgartigimod had just been officially listed in our country. After careful consideration and thorough communication, we have ultimately decided to attempt the use of efgartigimod in the hope of achieving rapid symptom relief by combining it with LPE.

FcRn is a multifunctional Fc-gamma receptors that can bind to circulating immunoglobulins, reducing their degradation in lysosomes and facilitating their release into the extracellular space (17). Therefore, inhibiting FcRn can increase the catabolism of immunoglobulins and autoantibodies, providing targeted treatment for immune-mediated diseases like MG (18). Efgartigimod, an FcRn antagonist, competitively binds to FcRn, and its clinical efficacy has been demonstrated in various randomized trials (6, 19, 20). Particularly, the global multicenter, randomized, double-blind, placebo-controlled study called ADAPT (6), which laid the foundation for efgartigimod’s approval, showed that in the first cycle, the proportion of MG-ADL responders in the efgartigimod group (68%) was significantly higher than that in the placebo group (30%). Additionally, there are now real-world data showing the effectiveness and safety of efgartigimod (21). Since efgartigimod was just approved in China in September 2023, there is still a lack of real-world data for Chinese MG patients. Furthermore, the specific indication for efgartigimod is generalized MG with AChR-Ab positivity. However, previous studies have included a small number of MuSK-MG patients, who have shown varying degrees of efficacy, despite their limited representation. Therefore, our patient’s positive response to efgartigimod undoubtedly brings new experiences for MuSK-MG cases that have shown poor response to conventional treatments. In addition, rozanolixizumab, another FcRn inhibitor, has demonstrated better efficacy for MuSK-MG in its Phase III study (22), indicating that FcRn is gradually becoming a powerful tool in the treatment of MuSK-MG. It is worth mentioning that although the latest German guidelines (23) state that rituximab is the first-line treatment for MuSK-MG and FcRn inhibitors are considered second-line treatment, when faced with patients experiencing acute exacerbations, utilizing FcRn inhibitors to rapidly decrease the levels of pathogenic antibodies appears to be a better choice. Therefore, our choice of efgartigimod seems to be quite reasonable. Fortunately, our patient did not progress to myasthenic crisis (MC). There has been case reported significant efficacy of efgartigimod in MC (24), indicating the endless potential of this novel biologic agent in the treatment of MG.

Efgartigimod can effectively reduce the IgG levels in patients’ body, which was evident from the notable decrease in IgG levels during the first four follow-up visits. Being a humanized IgG1 Fc fragment (25), efgartigimod is designed based on its high affinity between FcRn and IgG1 (26), so after competitive binding with FcRn, it leads to the maximum reduction in IgG1 levels. Although the pathological mechanism of MuSK-MG is mainly mediated by IgG4 (27), the ADAPT study found that efgartigimod reduces antibody levels to a similar extent across various subtypes (6). This is also one of the reasons why we chose efgartigimod for the treatment of this case of MuSK-MG. Interestingly, we also monitored the levels of MuSK antibodies, which did not show a consistent decrease like IgG levels, but always remained at higher levels and each follow-up result indicated >12.00 U/mL (tested via ELISA, reference value <0.40 U/mL). We speculate that this may be related to the high baseline levels of MuSK antibodies in the patient or limitations of the detection methods used. Fortunately, our patient showed significant improvement in clinical symptoms with four injections of efgartigimod. It’s worth noting that one month after the last dose, the patient’s IgG levels had started to rise significantly (**Figure 1**), but until the most recent telephone follow-up, more than two months after the last dose, the patient is still maintaining a good clinical condition (MG -ADL score 0). We speculate that although IgG levels were increasing, the levels of MuSK autoantibodies have not yet shown a significant rise, because previous studies have suggested that the concentration of MuSK antibodies appears to be correlated with the severity of the disease (28, 29). It should be noted that the clinical improvement in our case may involve additional effect of LPE. Therefore, we cannot attribute the patient’s recovery solely to the influence of efgartigimod. At present, the application of LPE in the treatment of MG is not widely performed. Based on the limited research on its effectiveness in MG treatment, it is observed that LPE appears to demonstrate significant clinical improvement in a short period of time. In the study conducted by Ouyang et al. (30), which included acute exacerbation MG patients treated only with LPE in addition to standard medication, a significant decrease in QMG and MG-ADL scores was observed as early as the first week after the first LPE. Duan et al. (14) compared the efficacy of LPE and PE in the treatment of MG, the score was completed within 3 days after treatment, and reported that the QMG score in the LPE group decreased by an average of 6.26 ± 4.39 points. However, in contrast, our patient’s scores in this case remained high after three LPE sessions, about one week after the first LPE. In light of this, although there may be a synergistic effect, we consider that the role of efgartigimod in this context is unquestionable. But larger cohort studies are still needed to provide more convincing evidence.

Our patient is currently not willing to maintain treatment further due to his good clinical condition. However, long-term maintenance treatment for MuSK-MG patients remains a challenging issue that we need to consider. Long-term use of corticosteroids often leads to various side effects, especially in elderly individuals. Compared to AChR-MG, there is limited evidence and poor efficacy for most non-steroidal immunosuppressive therapies such as tacrolimus, methotrexate, and azathioprine in MuSK-MG (31, 32). Before the advent of FcRn inhibitors, rituximab is a worthy option for MuSK-MG who are not satisfied with initial immunotherapy (33). In the ADAPT study (6), patients who completed the study or were unable to complete a cycle before study end were transferred to the follow-up open-label, single-arm, 3-year extension study (ADAPT+). Currently, in the interim analysis of ADAPT+, and the complete results of long-term follow-up presented by American Academy of Neurology (AAN), a total of 145 patients were included who received at least one cycle of efgartigimod treatment, with a maximum follow-up period of 3 years. Clinically significant improvements in MG-ADL and QMG scores were observed at each treatment cycle, with the majority of patients achieving a ≥2-point benefit in MG-ADL. The overall safety of efgartigimod was comparable to placebo (8). These findings suggest that long-term efgartigimod treatment further increases the proportion of patients achieving clinically meaningful improvement. We look forward to the publication of this study’s complete results and anticipate more research emerging to provide new directions for the long-term maintenance treatment of MG patients.

## 4 Conclusion

MuSK-MG has always been a challenging aspect in the treatment of MG, and the limited number of cases is a major reason for insufficient evidence in evidence-based medicine. For MuSK-MG patients who do not respond well to conventional treatments, it is worth further research to find more effective treatment options. As an emerging biologic agent, efgartigimod has shown a good momentum in the treatment of MG. Currently, there are no studies or cases systematically report the efficacy of efgartigimod in the treatment of acute exacerbations in MuSK-MG patients with poor response. Our case provides some clinical experience in this regard. However, the internal mechanism of MuSK-MG and long-term effective maintenance therapy for patients still need to be further explored.

## Data availability statement

The original contributions presented in the study are included in the article/supplementary material. Further inquiries can be directed to the corresponding authors.

## Ethics statement

Written informed consent was obtained from the individual(s) for the publication of any potentially identifiable images or data included in this article.

## Author contributions

GZ: Writing – review & editing, Writing – original draft, Data curation, Conceptualization. YM: Writing – review & editing, Investigation, Data curation. HZ: Writing – review & editing, Formal Analysis. XN: Writing – review & editing, Methodology. WQ: Writing – review & editing, Methodology. LH: Writing – review & editing, Supervision. XG: Writing – review & editing, Supervision, Conceptualization.
